# Coffee and beverages are the major contributors to polyphenol consumption from food and beverages in Japanese middle-aged women

**DOI:** 10.1017/jns.2014.19

**Published:** 2014-10-22

**Authors:** Yoichi Fukushima, Takeshi Tashiro, Akiko Kumagai, Hiroyuki Ohyanagi, Takumi Horiuchi, Kazuhiro Takizawa, Norie Sugihara, Yoshimi Kishimoto, Chie Taguchi, Mariko Tani, Kazuo Kondo

**Affiliations:** 1 Nestlé Japan Ltd, NYK Tennoz Bldg., 2-2-20 Higashi-Shinagawa, Shinagawa-ku, Tokyo 140-0002, Japan; 2 Japan Frozen Foods Inspection Corp., 2-4-6 Shiba-daimon, Minato-ku, Tokyo 105-0012, Japan; 3 Ochanomizu University, 2-1-1 Ohtsuka, Bunkyo-ku, Tokyo 112-8610, Japan

**Keywords:** Polyphenols, Consumption, Beverages, Food, Coffee, TP, total polyphenol

## Abstract

Food and beverages rich in polyphenols have been shown to reduce the risk of non-communicable diseases. The present study estimated polyphenol levels and consumption from food and beverages in Japanese women. Randomly recruited housewives living in the area around Tokyo (*n* 109; aged 21–56 years; Group 1) recorded all beverages and foods they ingested for 7 d, and the total polyphenol (TP) consumption was estimated based on the TP content of each item measured with a modified Folin–Ciocalteu method. For Group 1, TP was consumed at 841 (sd 403) mg/d (range 113–1759 mg/d), and beverages were a larger source of TP (79 %) than food (21 %). The largest single source of TP was coffee at 47 %, followed by green tea, black tea, chocolate, beer and soya sauce, at 16, 5·7, 3·3, 3·2 and 3·1 %, respectively. In terms of food groups, cereals/noodles, vegetables, fruits, beans and seeds, and seasonings (except for soya sauce) contributed 5·0, 4·0, 1·4, 1·8 and 2·4 %, respectively. Another group of housewives who consumed at least one cup of coffee per d were separately recruited (*n* 100; Group 2) in the same area. Their consumption of TP was higher at 1187 (sd 371) mg/d (range 440–2435 mg/d) than Group 1 (*P* < 0·001), and the difference mostly came from the coffee consumption. We conclude that not food but beverages, especially coffee, may be the major contributor to TP consumption in Japanese women.

Polyphenols, which exist ubiquitously in plants for protection against UV light and reactive oxygen species^(^^1^^–^^3^^)^ and also sometimes provide colours, are varied molecules containing over 8000 species, including flavonoids (for example, catechins in tea and cocoa, isoflavones in beans, quercetin in onions and anthocyanins in fruit) and non-flavonoids (for example, chlorogenic acids in coffee)^(^^4^^)^. Polyphenols are consumed by humans as a major part of non-nutrient food components. Total polyphenol (TP) content and antioxidative capacity are positively correlated (*R* 0·7–0·9)^(^^5^^,^^6^^)^, suggesting that dietary polyphenols are important and provide a large amount of antioxidants in the daily food intake. Polyphenols are foreign materials for humans and are metabolised and/or conjugated immediately after absorption and are excreted within 1 d. Some polyphenols, such as those from tea and coffee, are highly bioavailable; about 30 % are absorbed in the circulation in humans and their continuous consumption improves biomarkers for oxidative stress in humans^(^^7^^,^^8^^)^.

Coffee provides the highest proportion of antioxidants in the diet of the populations of some European countries, including Italy, Finland, France and Spain^(^^9^^–^^12^^)^ and is one of the beverages that has been most widely studied in epidemiological studies with respect to its health benefits. Meta-analysis of cohort studies has shown that adequate consumption of coffee may be beneficial to reduce the risk of type 2 diabetes^(^^13^^,^^14^^)^, total cancers^(^^15^^)^ and some specific cancers, including liver^(^^16^^)^ and endometrial cancers^(^^17^^)^, heart failure^(^^18^^)^, stroke^(^^19^^)^, Alzheimer's disease^(^^20^^)^ and Parkinson's disease^(^^21^^)^, resulting in reducing the risk of total mortality^(^^22^^)^. Coffee polyphenols, such as chlorogenic acids, are an important source of antioxidants^(^^23^^–^^25^^)^ in our daily life, and a high consumption of antioxidants from coffee may contribute to reducing mortality and morbidity risks. Coffee also has anti-inflammatory properties^(^^26^^,^^27^^)^ and is protective against oxidative damage^(^^8^^)^. Tea, which is rich in catechins, is also an important source of polyphenols and reduces the risk of type 2 diabetes^(^^13^^,^^28^^)^, some cancers^(^^29^^,^^30^^)^, stroke^(^^31^^)^ and CVD^(^^32^^)^. Not only coffee and tea, but also beverages, such as red wine, and foods, such as chocolate, fruit and vegetables, could be candidates as sources of polyphenols in the diet. Black chocolate and high-flavonoid cocoa have been shown to have beneficial effects on CVD^(^^33^^)^ and stroke^(^^34^^)^. Soya products containing isoflavones may reduce the risk of prostate cancer and breast cancer^(^^35^^,^^36^^)^. Total flavonoids may reduce the risk of type 2 diabetes^(^^37^^)^ and CVD^(^^38^^)^. Most of these epidemiological studies have shown an association between disease risks and consumption of the food itself, and it is not fully known how much polyphenols and/or antioxidants exert a beneficial impact.

The life expectancy of Japanese was the highest for women at 86·4 years, and is fifth for men at 79·9 years in 2013, and the Japanese diet may contribute to their healthy life. Several studies have reported on the consumption of TP in European and American countries^(^^10^^,^^12^^,^^39^^,^^40^^)^; however, information about polyphenol consumption by Japanese is limited. In our previous study^(^^5^^)^, we evaluated TP consumption from non-alcoholic beverages; however, information on food and alcoholic beverages was lacking, and there are no reports on polyphenol consumption from all food and beverages in the Japanese population. Adverse effects of polyphenol consumption from food and beverages in daily life are not known at present, and information is lacking about how high polyphenol consumption can be achieved in individuals. The present study aimed to estimate TP consumption among Japanese middle-aged females and to describe the contribution of specific food and beverage sources and their individual differences, in order to provide further information about polyphenol consumption and to understand their potential contribution for health benefits and their safety.

## Materials and methods

### Total polyphenol assays

TP content in food and beverages was measured using a modified Folin–Ciocalteu method^(^^41^^)^ described in a previous report^(^^5^^)^. Briefly, food and beverage samples were purchased in Japan. The fresh edible portions of foods were chopped and homogenised in an extraction solution, 70 % ethanol and 0·9 % NaCl (7:3, v/v), for 1 min and were sonicated for 10 min at 4°C, and then centrifuged at 3000 rpm for 5 min to obtain extracts. Beverages were extracted by acetone–water solution (7:3, v/v). The filtered and diluted solvent extract (SE) solutions were applied on an Oasis HLB cartridge (Waters Japan), which absorbed polyphenols, and a washing extract (WE) eluted solution was obtained. Folin–Ciocalteu reagent was added to each SE and WE solution and was incubated for 15 min at 50°C with sodium carbonate solution, after which specific absorbance at 760 nm was measured. The TP level was determined by subtracting the value of the WE solution, which contains interfering water-soluble components such as reducing sugars and ascorbic acid, from the value of the SE solution. Chlorogenic acid and catechin (Sigma-Aldrich Japan KK) were used as standards for coffee and other food and beverages, respectively. A total of seventy-seven food and beverage items were selected based on information of trade volume in the Japanese market and TP contents from literature^(^^42^^–^^45^^)^ and their TP levels were measured.

### Survey for food and beverage consumption

The survey was conducted in 2010. Housewives living in the area around Tokyo were randomly recruited and included 109 subjects aged 21–56 years (Group 1). Another group of housewives, who consumed at least one cup of coffee per d, were separately recruited in the same manner (*n* 100; Group 2). The profiles of all subjects are shown in Table 1. Subjects recorded all beverages and food materials consumed and the cooking menus used for 7 d. Consumption of foods and energy intake was calculated with nutrition-calculating software EXCEL EIYO-KUN (version 5·0; Kenpakusha), which provides portion sizes and weights of food materials for cooking menus^(^^46^^)^. TP consumption was estimated from the food and beverage consumption data and TP contents.

**Table 1.** Profile of subjects in the study (Mean values and standard deviations)

	Group 1 (recruited with no limitation)		Group 2 (subjects with coffee consumption)	
	Mean	sd	Mean	sd
Number of subjects	109		100	
Age (years)	38·3	7·5	39·3	5·6
Energy consumption				
kJ/d	7508	1616	7483	1406
kcal/d	1794	386	1788	336
Coffee consumption (ml/d)	195	142	401	151
Total non-alcohol beverage consumption (ml/d)	666	267	767	250
Total alcohol beverage consumption (ml/d)	121	206	81	142
Total food consumption (g/d)	1038	240	1031	209

All beverage items were recorded and are fully covered with TP content information. A total of eighty-two vegetable and thirty-six fruit items were recorded as consumed by all subjects in 7 d, and the weight-based coverage of consumption of vegetable and fruit items with information of TP contents was 94 % (thirty-one vegetables) and 95 % (eleven fruits). For the confectionery products, 42 % consumption weight came from chocolate and wheat-based cookies, which were used for calculation of TP consumption. Other confectionery products, such as rice cookies, sugar drops and other snacks, were recorded but excluded from the calculation because their variety was too large to estimate the TP contents and the expected amounts of TP were low.

### Statistical analysis

The results are presented as mean values and standard deviations. Data analysis was carried out with IBM® SPSS® Statistics 19 (SPSS Japan Inc.) using the Wilcoxon rank sum test. A difference between means is considered significant at *P* < 0·05.

## Results

TP levels were measured with a modified Folin–Ciocalteu method for seventy-seven food and beverage items, including thirty-one vegetables and potatoes, eleven fruits, eight cereals and noodles, five beans and seeds, five seasonings, two confectionery items, and twelve non-alcoholic and three alcoholic beverages (Table 2). The consumption of TP from foods and beverages by subjects is shown in Table 3. Consumption of TP by the subjects of Group 1, who were randomly recruited from the population living in the area around Tokyo (*n* 109; aged 38·3 (sd 7·5) years) was 841 (sd 403) mg/d. The between-subject variation in TP consumption were large and ranged from 113 to 1759 mg/d, where 15-fold differences were observed in subjects from Group 1. Beverages were a larger source of TP (79 %) than food (21 %) (Table 4). The largest source of TP was coffee at 47 % (0–86 %) followed by green tea at 16 % (0–66 %), then by black tea, chocolate, beer and soya sauce at 5·7 (0–50), 3·3 (0–28), 3·2 (0–53) and 3·1 (0·6–21) %, respectively. In food categories, cereals and noodles, vegetables, fruits, beans and seeds, and seasonings (except for soya sauce) contributed 5·0, 4·0, 1·4, 1·8 and 2·4 %, respectively. Total soya-based products, including soya milk and seasonings, contributed 5·9 % and total wheat-based products, including bread, pasta and udon noodles, contributed 3·7 %.

**Table 2.** Total polyphenols (TP; mg/100 g)* in foods and beverages (Mean values and standard deviations)

		Mean	sd
Vegetables and potatoes			
Red pepper		448·6	173·0
Dried laver seaweed		204·0	58·9
Parsley		156·8	23·5
Ginger		80·0	12·6
Burdock		49·3	9·3
Green chives		48·4	20·3
Onions		46·5	11·0
Spinach		41·6	13·7
Araceous		35·5	26·1
Broccoli		35·2	8·5
Green onions		32·5	15·6
Egg plant		30·7	2·8
Japanese mustard spinach		18·5	4·4
Sweet potatoes		17·7	6·1
Welsh onions		15·0	12·6
Radishes		13·2	10·6
Maize		13·2	11·5
Bamboo shoots		11·7	9·3
Green pepper		11·7	10·9
Bean sprouts		6·5	4·6
Tomatoes		5·8	6·7
Pumpkin		5·4	5·1
Cabbage		5·1	3·5
Chinese cabbage		4·5	3·1
Celery		3·6	3·1
Potatoes	<0·1		
Cucumber	<0·1		
Carrots	<0·1		
Lettuce	<0·1		
Yams	<0·1		
Konjak	<0·1		
Fruits			
Prunes (dried)		238·7	59·8
Strawberries		100·3	23·1
Sour plums		56·7	13·1
Grapefruit		55·6	4·2
Oranges		54·9	6·3
Bananas		19·2	6·8
Grapes		11·7	13·3
Persimmon		10·3	7·5
Apples		4·8	1·8
Pears		4·3	3·1
Melon		2·4	2·7
Cereals and noodles†			
Buckwheat noodles		110·7	20·3
Pasta (dry weight converted)*		37·8	8·1
Wheat		36·8	7·9
Bread		28·4	17·9
Brown rice		25·9	8·4
Chinese noodles*		15·0	3·2
Udon noodles*		10·7	2·3
White rice		0·8	1·1
Beans and seeds			
Sesame		298·4	151·0
Almonds		266·9	224·1
Natto		114·4	41·1
Tofu		32·8	0·5
Sweetened red bean paste		18·5	1·0
Seasonings			
Curry powder		891·5	168·0
Soya sauce		185·1	31·0
Pepper		147·8	68·0
Miso		121·5	14·0
Horseradish		35·9	17·8
Confectionery			
Chocolate‡		935·5	201·6
Biscuits*		24·2	5·2
Beverages			
Coffee (roast and ground)		236·8	18·2
Coffee (soluble)		214·0	13·0
Coffee (ready to drink)		142·0	9·9
Green tea		125·8	65·0
Black tea (ready to drink)		90·0	26·0
Tomato/vegetable juice		88·4	48·0
Black tea (brewed)		71·4	26·0
Cocoa drink		59·6	20·0
Soya milk		52·2	52·8
Chinese tea		39·4	5·0
Fruit juice		32·6	18·0
Barley tea		12·6	5·0
Alcoholic beverages			
Red wine		230·0	78·1
Beer		26·6	6·7
Sake	<0·1		

* Calculated from TP contents from wheat.

† TP contents of noodles are reported as mg/100 g wet weight except for pasta.

‡ Calculated from TP contents assuming that 12 % cacao mass is used for chocolate in Japan according to national statistics in 2011 (http://www.chocolate-cocoa.com/statistics).

**Table 3.** Polyphenol consumption from food and beverages in general housewives (Group 1) and those consuming coffee every day (Group 2) (Mean values and standard deviations)

		Group 1				Group 2			
		Daily intake (g/d)		Polyphenol intake (mg/d)		Daily intake (g/d)		Polyphenol intake (mg/d)	
Food and beverages	Total polyphenols (mg/100 g)	Mean	sd	Mean	sd	Mean	sd	Mean	sd
Cereals and noodles (total)	–	275·7	58·6	42·3	19·7	275·2	57·1	43·2	15·6
Bread	28·4	42·6	27·9	12·1	7·9	60·3	30·5	17·1	8·7
Buckwheat noodles*	110·7	7·6	14·9	8·4	16·5	2·9	8·6	3·2	9·5
Wheat	36·8	18·4	10·2	6·8	3·8	18·3	10·7	6·7	4·0
Pasta†	37·8	10·5	11·1	4·0	4·2	12·6	10·9	4·7	4·1
Udon noodles*	10·7	28·9	33·5	3·1	3·7	26·1	31·9	3·0	3·7
Rice	1·7	130·5	37·3	2·1	0·6	120·9	33·8	1·9	0·6
Vegetables and potatoes (total)	–	229·8	69·1	33·5	11·5	215·1	71·7	31·4	10·9
Onions	46·5	31·4	14·6	14·6	6·8	30·8	16·3	14·3	7·6
Radishes	13·2	21·7	17·0	2·9	2·2	15·5	14·1	2·0	1·9
Spinach	41·6	5·2	8·2	2·2	3·4	4·1	7·4	1·7	3·1
Ginger	80·0	1·8	2·4	1·4	2·0	1·3	1·2	1·1	1·0
Burdock	49·3	2·7	4·6	1·3	2·3	2·4	4·7	1·2	2·3
Araceous	35·5	3·4	5·9	1·2	2·1	1·3	3·5	0·4	1·2
Sweet potatoes	17·7	5·5	10·7	1·0	1·9	5·0	10·7	0·9	1·9
Fruits (total)	–	50·4	41·5	11·7	11·5	57·0	43·4	15·5	24·4
Oranges	54·9	10·7	15·8	5·8	8·7	8·6	15·4	4·7	8·5
Strawberries	100·3	1·4	2·8	1·4	2·8	1·2	3·2	1·2	3·2
Bananas	19·2	5·8	13·3	1·1	2·6	8·1	12·8	1·6	2·4
Beans and seeds (total)	–	30·1	21·8	15·5	11·6	36·1	25·3	19·0	13·9
Natto	114·4	5·9	7·8	6·7	9·0	8·1	9·8	9·3	11·3
Tofu	32·8	12·7	12·9	4·2	4·2	15·7	17·8	5·1	5·8
Other soya products (nine items)	–	6·4	7·3	1·8	2·0	6·2	7·4	1·8	2·4
Seasonings (total)	–	60·1	27·6	46·4	21·8	57·7	24·8	46·0	21·8
Soya sauce	185·1	14·3	5·5	26·5	10·2	12·5	5·4	23·1	10·0
Miso	121·5	8·6	12·9	10·4	15·7	9·1	11·7	11·0	14·2
Curry powder	891·5	0·3	0·3	2·3	2·5	0·3	0·3	2·5	2·9
Pepper	147·8	1·2	0·5	1·8	0·8	1·2	0·5	1·8	0·7
Confectionery (total)	–	7·7	8·4	27·5	41·6	10·3	8·9	34·3	42·0
Chocolate	936	2·9	4·4	27·5	41·5	3·7	4·5	34·2	42·0
Total food	–	895	191	177	60	854	169	188	63
Beverages (total)	–	778	324	664	385	822	301	999	342
Coffee	200	194·7	142·1	395·6	297·2	400·8	150·7	812·0	303·9
Green tea	115	120·6	146·4	137·7	170·4	74·1	108·2	83·7	129·6
Black tea	96	45·0	65·3	47·8	73·3	42·2	57·6	44·5	64·9
Oolong tea	39	37·1	99·2	14·5	38·7	27·9	83·8	10·9	32·7
Barley tea	9	126·0	154·8	13·7	18·5	90·1	130·3	9·2	13·6
Other teas	8	41·6	111·3	3·1	9·0	26·7	77·4	2·1	6·3
Cocoa/chocolate malt drink	62	4·2	16·6	2·6	10·3	2·8	8·5	1·8	5·3
Tomato/vegetable juice	69	7·5	24·1	5·2	16·6	8·8	29·1	6·1	20·1
Fruit juice	34	6·1	16·5	2·1	5·6	8·8	21·1	3·0	7·2
Soya milk	36	5·0	25·4	1·8	9·2	2·9	20·9	1·0	7·5
Beer	26·6	100·9	191·3	26·8	50·9	68·6	133·9	18·2	35·6
Red wine	230	5·9	22·8	13·6	52·6	2·8	9·6	6·5	22·1
Total food and beverages	–	1673	424	841	403	1676	392	1187	371

*Wet base.

†Dry base.

**Table 4.** Proportion and ranking of polyphenol consumption in general housewives (*n* 109)

Rank	Food and beverages	Type	% Consumption	% Cumulative consumption in rank	% Maximum consumption in individual
By categories					
1	Beverages	B	79·0	–	95·9
2	Seasonings	S	5·5	–	30·2
3	Cereals and noodles	C	5·0	–	29·3
4	Vegetables and potatoes	V	4·0	–	32·5
5	Confectionery	O	3·3	–	27·9
6	Beans and seeds	O	1·8	–	21·2
7	Fruits	F	1·4	–	15·0
By items					
1	Coffee	B	47·0	47·0	86·0
2	Green tea	B	16·4	63·4	65·5
3	Black tea	B	5·7	69·1	49·9
4	Chocolate	O	3·3	72·4	27·9
5	Beer	B	3·2	75·5	52·5
6	Soya sauce	S	3·1	78·7	20·6
7	Onions	V	1·7	80·4	13·7
8	Oolong tea	B	1·7	82·1	36·1
9	Barley tea	B	1·6	83·8	27·7
10	Red wine	B	1·6	85·4	31·9
11	Bread	C	1·4	86·8	14·0
12	Miso	S	1·2	88·1	11·0
13	Buckwheat noodles	C	1·0	89·1	14·0
14	Wheat	C	0·80	89·9	10·3
15	Natto	O	0·80	90·7	13·4
16	Oranges	V	0·70	91·4	9·7
17	Tomato/vegetable juice	B	0·61	92·0	20·4
18	Chinese noodles	C	0·57	92·5	11·8
19	Tofu	O	0·49	93·0	6·7
20	Pasta	C	0·47	93·5	5·5
21	Udon noodles	C	0·37	93·9	3·0
22	Other tea	B	0·37	94·3	16·6
23	Radishes	V	0·34	94·6	2·5
24	Cocoa/chocolate malt drink	B	0·31	94·9	16·4
25	Curry powder	S	0·27	95·2	3·0
26	Spinach	V	0·26	95·4	5·5
27	Rice	C	0·25	95·7	2·5
28	Fruit juice	B	0·25	95·9	18·9
29	Pepper	S	0·22	96·1	3·0
30	Soya milk	B	0·21	96·4	43·2
31	Ginger	V	0·17	96·5	1·3
32	Sesame	O	0·17	96·7	3·6
33	Strawberries	F	0·16	96·9	2·8
34	Burdock	V	0·16	97·0	2·7
35	Araceous	V	0·15	97·2	3·0
36	Bananas	F	0·13	97·3	1·9
37	Fried tofu	O	0·12	97·4	1·1
38	Sweet potatoes	V	0·12	97·5	2·1
39	Broccoli	V	0·11	97·6	1·4
40	Persimmon	F	0·10	97·7	0·9
41	Green onions	V	0·10	97·8	0·7
42	Tomato sauce	S	0·10	97·9	2·3
43	Green chives	V	0·10	98·0	3·0
44	Cabbage	V	0·09	98·1	1·1
45	Almonds	O	0·09	98·2	1·2
46	Apples	F	0·08	98·3	0·6
47	Egg plant	V	0·08	98·4	0·9
48	Sweetened read bean paste	O	0·07	98·5	0·9
49	Parsley	V	0·07	98·5	1·1
50	Tomatoes	V	0·07	98·6	0·7

B, beverage; S, seasoning; C, cereal; V, vegetable; O, other food; F, fruit.

We independently recruited another group of housewives who consumed at least one cup of coffee per d and allocated them to Group 2 (*n* 100; aged 39·3 (sd 5·6) years); their energy consumption was not different from that of the Group 1 subjects (Table 1). Consumption of TP was higher, at 1187 (sd 371) mg/d (range 440–2435 mg/d), in Group 2 than in Group 1 (*P* < 0·001) (Table 3). The contribution of TP from coffee reached 68 % in Group 2. TP consumption from green tea was slightly higher in Group 1 (138 (sd 170) mg/d) than in Group 2 (84 (sd 130) mg/d) (*P* = 0·020); however, the other food and beverage items for the two groups were not significantly different in polyphenol consumption.

**Fig. 1.**
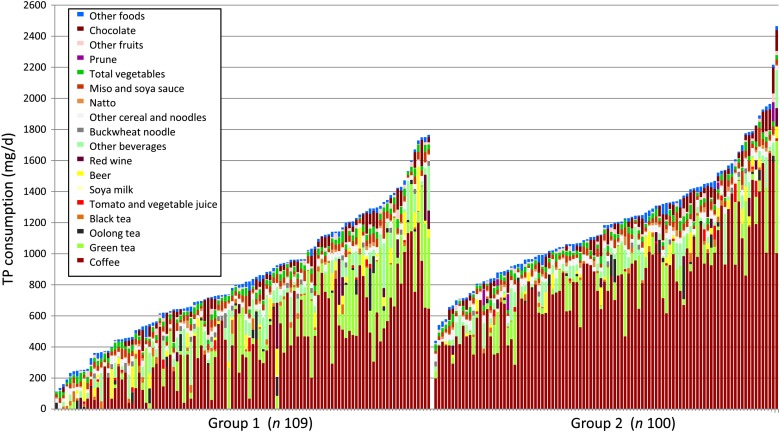
Personal consumption of total polyphenols (TP) from various foods and beverages by general housewives recruited without any limitation on coffee consumption (Group 1) and by housewives that consumed more than one cup of coffee per d (Group 2).

Personal consumption data from the two groups in this survey showed that seven items, including coffee, green tea, oolong tea, black tea, beer, red wine and chocolate, reached a maximum individual daily consumption of more than 200 mg/d in Group 1, which was at 1200, 608, 326, 272 mg/d, 285, 296 and 187 mg/d, respectively. Red wine and beer was consumed by fewer of the subjects but largely contributed to TP consumption in some subjects, accounting for up to 32 and 53 % of individual total TP consumption, respectively (Table 4 and Fig. 1). Three items, including tomato/vegetable juice, miso (fermented soya bean paste) and prunes, reached maximum individual daily consumption at 100–200 mg/d. Oolong tea, tomato/vegetable juice and soya milk also contributed to TP consumption in some subjects, accounting for up to 36, 20 and 43 % of TP consumption, respectively. Dried prunes were consumed by four subjects in Group 2, and its impact was high on TP consumption, reaching 102 mg/d (12·5 %) in one subject. In individuals, the largest source of TP was coffee in seventy-nine subjects (72 % of all subjects) in Group 1, followed by green tea (11 %), black tea (6 %), beer (3 %), red wine (2 %) and oolong tea (2 %). In Group 2, coffee was the largest source of TP in 99 % subjects, followed by green tea at 1 %.

## Discussion

This is the first study to show consumption of TP from both food and beverages in Japanese subjects. We selected housewives as subjects because recording all food materials with a cooking menu is a complicated task requiring experience of every-day cooking in order to maintain accuracy. The proportion of each beverage intake in subjects of the present study was similar to that found in our previous report^(^^5^^)^. Fruit and vegetable consumption was also close to the market statistics in Japan^(^^42^^)^, and food and beverage consumption in the two groups of the present study was mostly the same except for their coffee and green tea consumption. This implies that the assumption of TP consumption in the present study is not biased, even if the number of subjects and their representation are limited. TP consumption from the entire diet in the female Japanese subjects shown in the present study was 841 mg/d, and beverages were a four times larger source than foods. TP consumption from beverages, 664 mg/d, was slightly lower in the present study population compared with a previous study that reported 853 mg/d in Japanese men and women^(^^5^^)^.

Coffee was the largest source of polyphenol consumption, accounting for 49 % from all foods and beverages and 59 % from beverages in the subjects, which corresponds to our previous study showing that coffee is the largest source of TP in beverages at 50 %. Green tea was the second largest source of polyphenols at 16 %, whose proportion was slightly lower than that found in our previous study. There were no food items, except for coffee and green tea, that contributed an average of more than 10 % of TP consumption. In food categories, cereals/noodles, vegetables and seasonings made slightly higher contributions to TP at about 5 % of all TP consumption. Fruits had one-quarter less impact than the others. Chocolate and soya sauce were two major sources of polyphenols from food items, contributing 3 % of polyphenol consumption, which was twice as high as onions, which was the largest source from vegetables. In Group 2, where coffee consumption was at least one cup per d, 68 % of TP consumption came from coffee, and coffee significantly raised the overall TP consumption by 350 mg/d more in this group. The total consumption weight of foods and beverages was not different in the two groups, which suggests that the contribution of coffee to TP is large and that other sources did not compensate for the increase in TP by coffee. Subjects in Group 2, with higher coffee consumption, showed a slightly higher consumption of bread, yogurt and bananas, which are prone to be consumed at Western-style breakfasts in Japan. Their diets were lower in buckwheat noodles, radishes, Chinese cabbage, araceous and fish, which are mainly used for Japanese dishes, implying that coffee consumption may be associated with food choices. However, the differences in these amounts are not large and there are no differences in the total consumption of foods from each category.

Individual differences in TP consumption were large and we observed that subjects with the highest polyphenol consumption had a 15-fold higher consumption than the subjects with the lowest polyphenol consumption. Food and beverages whose personal TP consumption was at least 100 mg/d other than coffee and green tea were red wine, oolong tea, black tea, tomato/vegetable juice, beer, prunes, miso (fermented soya bean paste) and chocolate, which contributed a large amount of polyphenol consumption in some individuals. The major source of polyphenols was different in some individuals. Although coffee was the largest source of polyphenols in 72 % of the subjects, green tea, black tea, beer, red wine and oolong tea were also the largest source in some subjects. Most subjects (99 %) consumed coffee as the largest source of polyphenols among the subjects from Group 2 who consumed at least one cup of coffee per d.

TP consumption from both food and beverages described in currently available literature is summarised in Table 5^(^^10^^,^^12^^,^^39^^,^^40^^)^. These reports estimated that the daily TP consumption from foods and beverages was 800–1200 mg, which is a similar level to what we show in the present study. These studies show that beverages are a large source of polyphenols, and that coffee contributes the highest amount of polyphenol, about 50 % of TP. Tea is the second largest contributor; however, its contribution is rather lower than in Japanese. On the contrary, contributions from fruits, vegetables and cereals, which are followed by coffee and tea, are higher in European subjects than in Japanese subjects. The estimated polyphenol consumption from fruits and vegetables is also higher in the USA than in the present study^(^^43^^,^^47^^)^, where the contribution of apples and potatoes is high, which is similar in France^(^^48^^)^. Edible parts of fruits and vegetables are limited in Japan, where the peels of apples and potatoes, which are rich in polyphenols, are normally removed completely before consumption in Japan, which may cause the low content of polyphenols in the present study for these fruits and vegetables. The major cereal in Japan is white rice, which contains lower polyphenol levels than wheat products, and consumption of whole-grain cereal is also limited. Soya products are a typical contributor of polyphenol consumption in the Japanese diet, and soya-based seasonings, such as soya sauce and miso paste, which all Japanese generally use in their every-day diet, contributed to raise the polyphenol consumption equally in all Japanese. Recently, a database for polyphenols and/or flavonoids has been established in the USA, Europe and Asia^(^^49^^–^^52^^)^, and has been used for epidemiological studies that provide important knowledge of how polyphenols contribute to human health^(^^53^^–^^56^^)^. Polyphenol consumption in Japan and Europe is similarly high and beverages, especially coffee, are a major source; however, other types of contributing foods, especially fruits and vegetables, are different in various regions, where polyphenol contents as well as consumption differ. This implies that such a database is useful but it may require a careful integration of quantitative data, considering such regional differences.

**Table 5.** Estimated total polyphenol consumption in several countries*

							% Contribution							
Reference	Country	Subject number and age	Sex	Analytical method	Type of food (items)	Total polyphenol consumption (mean (sd); mg/d)	Beverages	Fruits	Vegetables	Cereals	Chocolates	Beans and seeds	Seasoning	Major source (% contribution)
Saura-Calixto *et al.* (2007)^(^^39^^)^	Spain	6000	–	Folin	Food and beverages (72*)	1106 (16)*	55	12	9	16	–	7	1	–
Ovaskainen *et al.* (2008)^(^^10^^)^	Finland	2007; 25–64 years	M, F	HPLC	Food and beverages (110)	863 (415)	74	9	3	12	0·04	–	–	Coffee (63), tea (9), berries (4), potatoes (2)
Pérez-Jiménez *et al.* (2011)^(^^12^^)^	France	4942; 45–60 years	M, F	HPLC	Food and beverages (452)	1193 (510)	63	17	7	4	6	0·7	0·03	Coffee (44), tea (9), apples (6), red wine (6), dark chocolate (6)
Zujko *et al.* (2012)^(^^40^^)^	Poland	6661; 20–74 years	M, F	Folin	Food and beverages (96)	1147	41	18	21	14	3	3	–	–
Fukushima *et al.* (present study)	Japan	109; 21–56 years	F	Folin	Food and beverages (77)	849 (403)	79	1	4	5	3	2	6	Coffee (47), green tea (16), black tea (6), chocolate (3), beer (3), soya sauce (3)
Fukushima *et al.* (2009)^(^^5^^)^	Japan	8768; 10–59 years	M, F	Folin	Beverages only (10)	853 (512)	–	–	–	–	–	–	–	Coffee (50), green tea (34), black tea (7), oolong tea (4), barley tea (2)

M, male; F, female.

* Food items were pooled into seven food groups for analysis. Extractable polyphenols were expressed to compare with the other studies.

There are two major methods used to measure polyphenol contents: the Folin and HPLC methods. The French and Finnish studies listed in Table 5 used the HPLC method to estimate TP consumption in their diets. HPLC is an accurate and quantitative method for measuring polyphenol molecules when standard samples exist. However, it is difficult to sum up all polyphenols in the diet and this may cause an underestimation of TP consumption in the diet. The Folin method is an easy analytical method that is suitable for roughly estimating overall amounts of polyphenols and the risk of underestimation is low. The Spanish and Polish studies listed in Table 5 and the present study used this method. For accuracy, the Folin method requires the avoidance of interference by reducing compounds, such as vitamin C, in the food samples^(^^57^^)^. In the present study, we used a modified Folin–Ciocalteu method with reverse-phase column chromatography to remove interference by reduced compounds, including vitamin C^(^^41^^)^. Coffee, cocoa and black tea, which provide a large part of the polyphenols in the diet, contain a large number of unknown phenolic compounds including polymers generated through roasting and/or fermentation processes. In the case of coffee, chlorogenic acids with nine major compounds (for example, mono- and dicaffeoyl quinic and feluoyl quinic acids) are composed of roughly one-third of the TP in roasted coffee^(^^5^^,^^58^^)^. Black tea polyphenol has two International Organization for Standardization (ISO) methods for its quantification, and a one-third difference is observed between the HPLC and Folin methods^(^^59^^)^. The choice of a chemical standard required for the Folin method may provide a slight difference in analytical results, and we used two major standards, chlorogenic acid for coffee and catechin for others, where the gradient of standard curves for commercially available polyphenol compounds, such as epigallocatechin gallate, epicatechin, epicatechin gallate, epigallocatechin, tannic acid, naringenin, and ethyl gallate, are within 2 sd difference with catechin except for chlorogenic acid^(^^5^^)^.

*In vitro* antioxidant capacity is used as an indicator to show the potential health benefits of foods^(^^49^^,^^60^^)^. Coffee is the largest contributor of antioxidant capacity in Spain and Italy^(^^9^^,^^11^^)^ but is low in the USA and was excluded in the Swedish study^(^^60^^,^^61^^)^, where inconsistencies may be caused by methodological differences in antioxidant capacity^(^^62^^)^. Each polyphenol is not equally bioavailable, which may also cause different impacts of polyphenols on health benefits. A human intervention study showed that TP in the urine is more predictive for all causes of death of the elderly than is its amount of oral consumption^(^^53^^)^, suggesting that the amount of polyphenol in the circulation is more meaningful for health benefits than is oral consumption, at least to some extent. Although antioxidant capacity or bioavailability are important to understand the actual impact of polyphenols exerting health benefits, TP content and its consumption can be a good practical indicator of potential health benefit in human life. Approximately one-third of orally consumed polyphenols is transferred into the circulation and can be identified as metabolites both for coffee and green tea^(^^63^^)^. These two beverages are major sources of polyphenols, providing two-thirds of TP consumption by the subjects. We found in the present study that the amount of polyphenols consumed has large individual differences. An indication of their contents could provide significant information for potential health benefits, especially in populations with low consumption of polyphenols, who may reduce their opportunity to be healthier.

In conclusion, the present study characterised TP consumption from all foods and beverages in middle-aged female subjects in Japan. Not food but beverages, especially coffee, were the largest source of polyphenols in the subjects' daily life, and large individual differences in polyphenol consumption were observed. These results are beneficial to understand healthy diets and polyphenol consumption as a potential contributor to that. Further studies are required to show TP consumption in different sex and age groups, and the relationship between polyphenol consumption and its health benefits.
